# A Patient-Oriented App (ThessHF) to Improve Self-Care Quality in Heart Failure: From Evidence-Based Design to Pilot Study

**DOI:** 10.2196/24271

**Published:** 2021-04-13

**Authors:** Constantinos Bakogiannis, Anastasios Tsarouchas, Dimitrios Mouselimis, Charalampos Lazaridis, Efstratios K Theofillogianakos, Antonios Billis, Stergios Tzikas, Nikolaos Fragakis, Panagiotis D Bamidis, Christodoulos E Papadopoulos, Vassilios P Vassilikos

**Affiliations:** 1 Cardiovascular Prevention and Digital Cardiology Lab Third Cardiology Department Aristotle University of Thessaloniki Thessaloniki Greece; 2 Third Cardiology Department Aristotle University of Thessaloniki Thessaloniki Greece; 3 Lab of Medical Physics School of Medicine Aristotle University of Thessaloniki Thessaloniki Greece

**Keywords:** mHealth, heart failure, smartphone app, self-care, COVID-19, patients, caregivers

## Abstract

**Background:**

Heart failure (HF) remains a major public health challenge, while HF self-care is particularly challenging. Mobile health (mHealth)–based interventions taking advantage of smartphone technology have shown particular promise in increasing the quality of self-care among these patients, and in turn improving the outcomes of their disease.

**Objective:**

The objective of this study was to co-develop with physicians, patients with HF, and their caregivers a patient-oriented mHealth app, perform usability assessment, and investigate its effect on the quality of life of patients with HF and rate of hospitalizations in a pilot study.

**Methods:**

The development of an mHealth app (The Hellenic Educational Self-care and Support Heart Failure app [ThessHF app]) was evidence based, including features based on previous clinically tested mHealth interventions and selected by a panel of HF expert physicians and discussed with patients with HF. At the end of alpha development, the app was rated by mHealth experts with the Mobile Application Rating Scale (MARS). The beta version was tested by patients with HF, who rated its design and content by means of the Post-Study System Usability Questionnaire (PSSUQ). Subsequently, a prospective pilot study (THESS-HF [THe Effect of a Specialized Smartphone app on Heart Failure patients’ quality of self-care, quality of life and hospitalization rate]) was performed to investigate the effect of app use on patients with HF over a 3-month follow-up period. The primary endpoint was patients’ quality of life, which was measured with the Kansas City Cardiomyopathy Questionnaire (KCCQ) and the 5-level EQ-5D version (EQ-5D-5L). The secondary endpoints were the European Heart Failure Self-care Behavior Scale (EHFScBS) score and the hospitalization rate.

**Results:**

A systematic review of mHealth-based HF interventions and expert panel suggestions yielded 18 separate app features, most of which were incorporated into the ThessHF app. A total of 14 patients and 5 mHealth experts evaluated the app. The results demonstrated a very good user experience (overall PSSUQ score 2.37 [SD 0.63], where 1 is the best, and a median MARS score of 4.55/5). Finally, 30 patients (male: n=26, 87%) participated in the THESS-HF pilot study (mean age 68.7 [SD 12.4] years). A significant increase in the quality of self-care was noted according to the EHFScBS, which increased by 4.4% (SD 7.2%) (*P*=.002). The mean quality of life increased nonsignificantly after 3 months according to both KCCQ (mean increase 5.8 [SD 15] points, *P*=.054) and EQ-5D-5L (mean increase 5.6% [SD 15.6%], *P*=.06) scores. The hospitalization rate for the follow-up duration was 3%.

**Conclusions:**

The need for telehealth services and remote self-care management in HF is of vital importance, especially in periods such as the COVID-19 pandemic. We developed a user-friendly mHealth app to promote remote self-care support in HF. In this pilot study, the use of the ThessHF app was associated with an increase in the quality of self-care. A future multicenter study will investigate the effect of the app use on long-term outcomes in patients with HF.

## Introduction

Heart failure (HF) is a major burden on patients, negatively affecting their functional status and quality of life. The incidence of HF in the United States is estimated between 2 and 5/1000 person-years [1]. There are about 26 million patients with HF globally [2]. HF decompensations occur frequently and necessitate lengthy hospital stays [3-5], while the disease causes mortality comparable with many types of cancer [6].

Managing HF is a difficult task for clinicians, and even more so for patients, as complicated and lengthy self-care is needed. Pharmacological therapy for HF consists of several different medications with different dosing strategies, especially in patients with HF with reduced ejection fraction (HFrEF) [7]. Diuretics for avoiding fluid congestion work optimally when the dosage varies depending on clinical or imaging indicators of fluid accumulation. Experienced patients often make such adjustments themselves successfully [8,9]. When medications for frequently occurring comorbidities, such as atrial fibrillation, hypertension, and diabetes, are also taken into account, the result is a labyrinthine, ever-changing medication regimen that requires time, presence of mind, and dedication to successfully adhere to [10,11].

Lifestyle changes are also a necessary but difficult part of HF self-care. Tracking fluid intake [9], daily weighing [8], increasing physical activity [12-14], and getting vaccinations [15,16] have all been shown to improve outcomes in HF, but sustained long-term adherence to the “HF lifestyle” is almost impossible without repeated interventions by a multidisciplinary team. This usually consists of physicians, nurses, dietitians, psychologists, and exercise physiologists [17].

Mobile health (mHealth) pertains to the use of mobile communications and network technologies for health care [18]. mHealth-based implementations can be designed for use by clinicians [19], nurses, allied health professionals, caregivers, or even the patients themselves [17,20]. The medium by which mHealth is delivered to the end user used to be mobile phone technologies such as automated phone calls and SMS text messages [21], but now has mostly migrated to newer technologies, such as smartphones and tablets [22-25]. The data from different types of devices including wireless scales, blood pressure monitor, or wearables (eg, sensors, bands) can be easily incorporated into such systems [26].

Patient-centered interventions utilizing mHealth technology already show promising results in improving the quality of self-care in several chronic diseases where patient participation is important, such as diabetes [27], hypertension [28], and depression [29]. The central pillar of such interventions is usually an app that provides patient education, encourages behavior that is appropriate for each disease (eg, salt restriction in HF), reminds the patient for actions that need to be taken (eg, medication/vaccination reminders), and potentially enables synchronous or asynchronous communication with health care personnel, caregivers, or even other patients with the same disease [17,22,23,25,30-32]. Remote monitoring is also a very appealing prospect [33].

In the setting of the COVID-19 pandemic, patients with severe comorbidities have a significantly higher risk of severe or even deadly disease progression [34]. It is thus of paramount importance that these patients are shielded from exposure to the virus [35]. In this situation, mHealth solutions for the remote monitoring and care of these patients may indeed become a crucial step in safeguarding this fragile group of patients. Coronavirus or not, HF, with all its intricate self-care, appears to be a prime target for mHealth-based interventions. Nonetheless, designing an app for use by patients with HF may prove challenging, as their particular needs and hindrances (eg, the mild cognitive decline HF is associated with) need to be considered.

**Figure 1:**
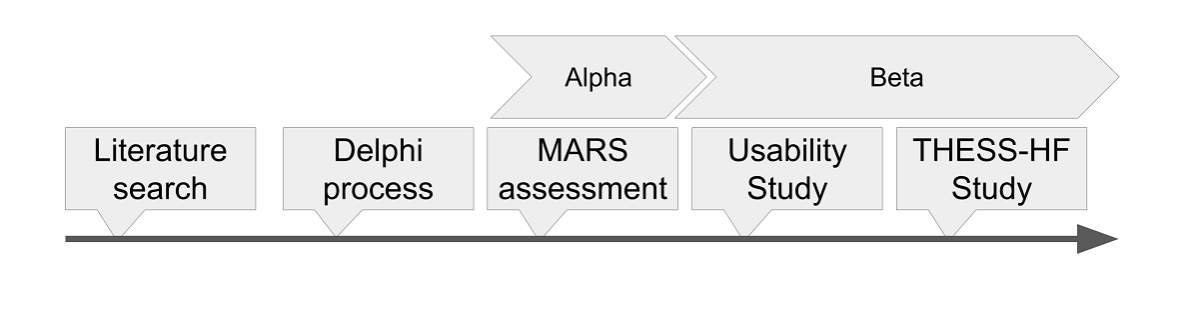
A timeline of the various parts of the evidence-based app development process, along with concurrent app versions. Note that the timeframes are not represented to scale in this figure.

Our aim was the development of an evidence-based, patient-oriented mHealth app (The Hellenic Educational Self-care and Support Heart Failure app [ThessHF app]) in cooperation with patients and their caregivers from our department’s Heart Failure Outpatient clinic. Furthermore, we aimed to assess ThessHF app functionality with experts as well as patients with HF in real-world settings. Finally, we conducted THESS-HF, THe Effect of a Specialized Smartphone app on Heart Failure patients’ quality of self-care, quality of life and hospitalization rate, a prospective study that investigated the clinical effect of app use on patients’ quality of life and hospitalization rate. A visual representation of this process is presented in Figure 1.

## Methods

### Development of a Patient-Oriented mHealth App: The ThessHF App

A systematic review of available literature was the first step in developing the app, as it was expected to reveal app features and design considerations that would increase the app’s usability and benefit to patients with HF. Our search focused on mHealth interventions that utilized smartphone technology; the results were codified as discrete app features that could be incorporated into the ThessHF app. These features were disseminated to a panel of 8 cardiologists and 2 physicians with mHealth familiarity. Furthermore, these features were discussed with patients with HF and their opinion was sought.

A 3-step Delphi process was used to reach a consensus on which app features were critical, optional, or indeed unsuitable for a patient-oriented HF app. After a consensus was achieved, physicians with programming experience then developed a beta version of the app. Throughout the app’s development process, patients with HF and caregivers were frequently called upon to give unstructured feedback. Prior to the app’s rollout in the usability study, 5 independent mHealth experts were given access to the ThessHF app for a month and invited to rate it using the Mobile Application Rating Scale (MARS) [36].

### Usability Study of the App

Patients that visited the HF clinic were invited to install the beta version of the app to their own Android smartphones for usability testing. Patients were serially recruited from the HF clinic, with exclusion criteria being not owning a smartphone, not understanding written Greek, and denying participation. No incentive was given to participate in the usability study. After a short 30-minute hands-on session in which the researchers trained patients in the app’s use, they were free to use it in any way they saw fit. A telephone number was given to them, which they could call for technical assistance or to report bugs. After a month of in-the-wild use of ThessHF, they evaluated the app by filling out the Post-Study System Usability Questionnaire (PSSUQ). The PSSUQ is a 16-item questionnaire that measures users’ perceived satisfaction of a system. PSSUQ scores can range from 1 to 7, with lower scores being better [37]. Its questions can be divided in 3 subdomains: system usefulness, information quality, and interface quality.

### The Effect of a Specialized Smartphone App on Heart Failure Patients’ Quality of Self-Care, Quality of Life, and Hospitalization Rate of Patients with Heart Failure (THESS-HF)

To examine the clinical effectiveness of the ThessHF app, we designed the “THe Effect of a Specialized Smartphone app on Heart Failure patients’ quality of self-care, quality of life and hospitalization rate” (THESS-HF) study. The THESS-HF study is a single-center, prospective study that recruits patients with HFrEF who own smartphones. Because the study took place largely during the COVID-19 pandemic, it was designed to obviate the need for physical visits, with all questionnaires and patient contact in general happening via telephone, instant messaging, or video conference calls. Patients were serially recruited from our department’s HF clinic. Patients should have HFrEF to qualify for inclusion in the study. Exclusion criteria were cognitive or visual impairment (defined as Montreal Cognitive Assessment score <20 and visual acuity worse than 20/50, respectively), a history of stroke in the preceding 12 months, and experiencing uncontrolled psychiatric diseases. No incentive was given to participate in the study.

At baseline, HF-specific quality of life was quantified using the Kansas City Cardiomyopathy Questionnaire (KCCQ) [38], whereas health-related quality of life was quantified with the 5-level EQ-5D version (EQ-5D-5L) [39]. Quality of self-care was measured with the European Heart Failure Self-care Behavior Scale (EHFScBs) [40]. Patients then received remote instruction regarding the installation and use of the ThessHF app. Patients were then followed up on for 3 months in total. After 3 months of app use, the KCCQ, EQ-5D-5L, and EHFScBs questionnaires were once again administered. The study’s primary endpoints were patients’ HF-specific and health-related quality of life, as quantified via the KCCQ’s total test score (KCCQ-TTS, ranging from 23 to 100, where 100 represents best HF-related quality of life) and the EQ-5D-5L visual analog scale score (EQ-5D-5L VAS, ranging from 0 to 100, where 100 represents the best health-related quality of life), respectively. Secondary endpoints were the quality of self-care score (EHFScBs, ranging from 0 to 100, where 100 represents the best quality of self-care) as well as the rate of hospitalizations or ER visits for HF decompensation during the follow-up period.

The study was approved by the local Institutional Research Board. The procedures followed were in accordance with the Helsinki Declaration of 1975, as revised in 2000.

### Statistical Analysis

Normally distributed continuous variables are reported as mean (SD) in text. Non-normally distributed continuous variables are reported as median (interquartile range). Normality was examined with the Kolmogorov–Smirnov test. The paired *t* test was used when comparing means between samples for normally distributed values, while the Mann–Whitney *U* test was used for comparing means between non-normally distributed values. The paired *t* test was used when evaluating changes in parameters for significance. Statistical significance was defined at a level *P*<.05. Spearman rho was used when looking for correlation between continuous or discrete variables.

## Results

### Feature Selection and App Development

Our search yielded 4 studies [22-25] that measured the effect of app-based interventions in patients with HF. The app features extracted from the literature review as presented in Table 1 were compiled and presented to the panel of experts participating in the Delphi process (a detailed review description of the systematic review is presented in Multimedia Appendix 1). The result of the process, as described in the “Methods” section, was the creation of a list of 18 critical, optional, and unsuitable/unnecessary app features (Table 2). The authors decided to declare active physician involvement a priori unsuitable, as such a feature would measurably compromise the scalability and cost-effectiveness of any mHealth intervention that used the ThessHF smartphone app.

**Table 1:** An overview of the 4 randomized controlled trials included in the systematic review.

Studies	Patients randomized to control or intervention group, study duration	App features	Hospitalizations	Quality of life	Notable outcomes	Comments
Seto et al [22]^a^	50/50, 6 months	Blood pressure and weight daily (F^b^) Once a week 1-lead electrocardiography (F) -On-call doctor can be contacted through the app (F) -Active doctor involvement in setting weight targets (N^c^)	No difference observed between groups (*P*=.1)	MLHFQ^d^ utilized Improved in the intervention group versus control (*P*=.05)	More patients were prescribed with aldosterone antagonists in the intervention group (*P*=.02)	Relatively outdated smartphone technology was utilized Visits to the HF^e^ clinic and nurse workload were increased disproportionately to outcomes
Vuorinen et al [23]^a^	47/47, 6 months	Only buttons are used in user interface (N) Blood pressure, heart rate, and weight daily (F) Assessment of dizziness, dyspnea, palpitations, weakness, edema (F) Measurement history as graphs (F) Active nurse involvement in monitoring patient data, encouraging app use (N)	No difference observed between groups (*P*=.35)	N/A^f^	More uptitration events of angiotensin-converting enzyme inhibitor/beta blocker medication (*P*=.04) and downtitration of diuretics (*P*=.02) in the intervention group versus control Medical staff’s (nurses) time spent for the intervention group was significantly greater versus control (*P*<.001)	No difference between groups regarding N-terminal pro-brain natriuretic peptide (NT-proBNP), left ventricular ejection fraction, and other clinical variables
Hägglund et al [24]^g^	40/32, 3 months	Body weight via wirelessly connected scale daily (F) App-directed diuretics titration (F) Visual analog scale assessment (N) App-directed alert to contact HF center via phone (F) Patient education module (F)	2.2 less hospital days per patient due to HF for the intervention group versus control (relative risk 0.38; *P*<.05)	KCCQ^h^ and SF-36^i^ utilized KCCQ showed significant improvement in the intervention group versus control (*P*<.05)	N/A	Data were stored in the tablet The intervention included patient education and advices regarding self-care in adherence to the guidelines for HF (eg, consult for increase in diuretics if body weight gain detected)
Athilingam et al [25]^a^	9/9, 1 month	Body weight daily (F) Symptom assessment daily (F) Heart rate and activity monitoring via chest strap (F) Medication tracker and reminder (F) Patient education module (F) Deep breathing and walking exercises (F)	N/A	KCCQ utilized No statistical difference	Significant improvement regarding self-care management (*P*=.01) and self-care confidence (*P*=.03) of the intervention group versus the control group, as appraised by the Self-Care of Heart Failure Index	Small sample size Only 72% of the patients concluded the 30-day follow-up Patients stated their preference for the use a smartphone app alone or combined with a wrist wearable tracker over the chest strap

^a^The study intervention was smartphone based.

^b^Denotes functional features (ie, those directly specific to patient self-management).

^c^Denotes nonfunctional features (ie, those not directly specific to patient self-management features).

^d^MLHFQ: Minnesota Living with Heart Failure Questionnaire.

^e^HF: heart failure.

^f^N/A: not applicable.

^g^The study intervention was tablet based.

^h^KCCQ: Kansas City Cardiomyopathy Questionnaire.

^i^SF-36: 36-Item Short Form Survey.

**Table 2:** App features evaluated by a panel of 8 cardiologists and 2 physicians with mHealth expertise.

Critical	Optional	Unnecessary/unsuitable
App-directed alert to contact heart failure center/medical personnel Blood pressure measurement Body weight measurement Buttons-only user interface Medication reminder Patient education module	Activity tracking Active nurse involvement in monitoring patient data Gamification features^ab^ Measurement history as graphs^b^ Pulses measurement^b^ Symptom assessmentb (eg, dizziness, edem^a^) Blood glucose measurements in diabeticsa,^b^	Active doctor involvement in monitoring parameters/reacting to emergency calls App-directed diuretics titration Chest strap One-lead electrocardiography Visual analog scale assessment Wireless weighing scale

^a^Features not extracted during the systematic review.

^b^Optional features introduced in the ThessHF app.

### The ThessHF App

**Figure 2:**
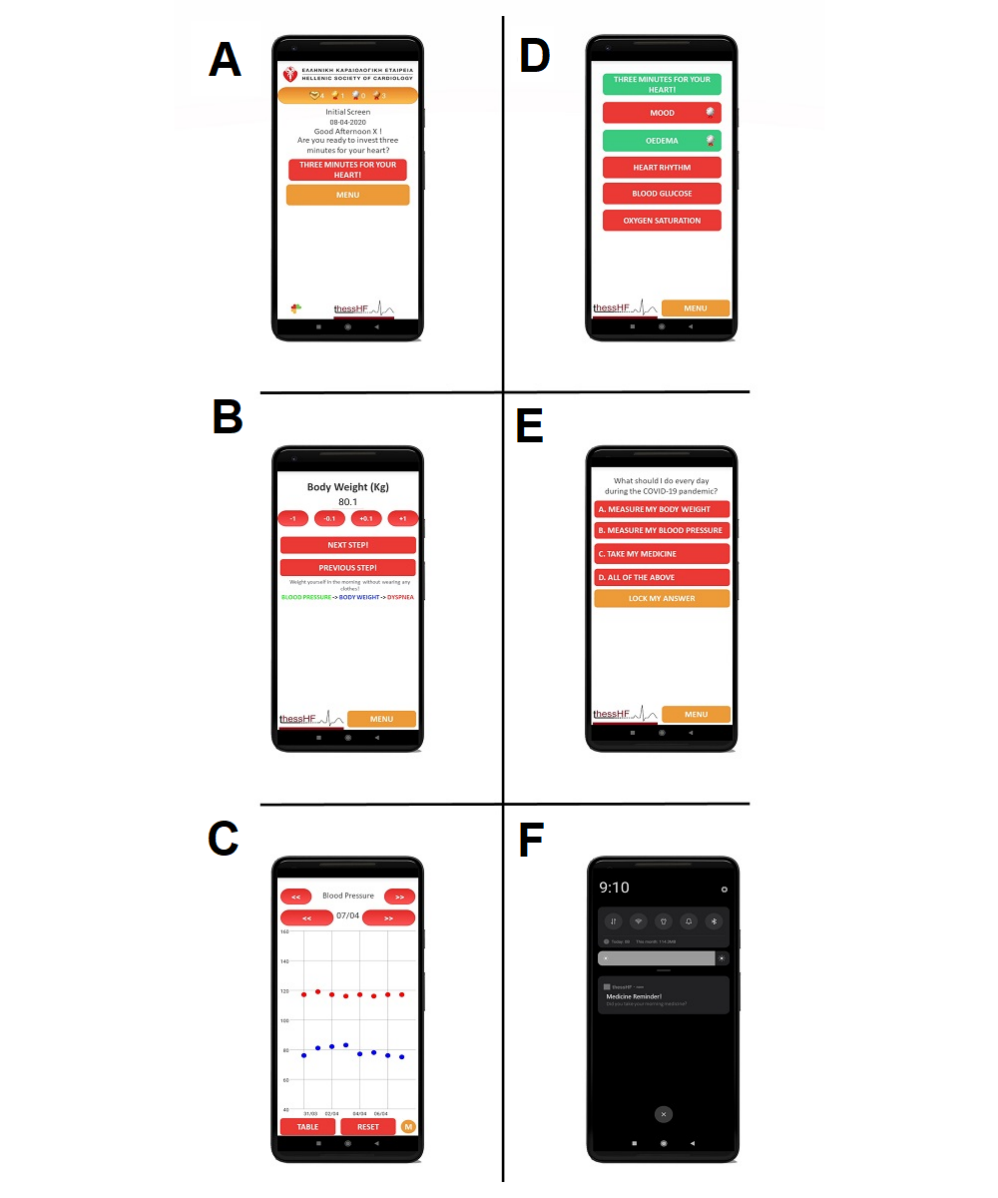
Screenshots of the ThessHF app translated in English, displaying (A) the main screen and gamification features, (B) the activity where patients input their blood pressure, (C) history of blood pressure available as a graph, (D) the main menu screen, where inputted parameters turn green, (E) a question out of the weekly quiz, and (F) the push notification reminding patients to take their pills.

Physicians developed the ThessHF app, incorporating all “critical” and most “optional” features (Table 2) in the beta version. The app encourages patients to invest 3 minutes daily to perform necessary self-care steps (weigh themselves, measure their blood pressure, and quantify potential dyspnea) and log the results in the app. Values that lie outside the ranges predetermined by clinicians alert the patient to seek medical help, while a timeline of the aforementioned parameters is always available. Patients are also reminded to take their medication in the morning, afternoon, and evening. Patient education is included in the form of a weekly quiz that contains questions about the disease. In an effort to achieve long-term adherence to the app, gamification features have been implemented, rewarding patients with medals as they interact with them. The app features weight and symptoms tracking, medication reminders, gamification features, and a weekly HF quiz for educational purposes (Figure 2, Multimedia Appendix 2). A translated version of the text found in the app is presented in Multimedia Appendix 3. With regard to the app evaluation by the mHealth experts with the MARS, the app received a median score of 3.80 regarding user engagement, 4.0 for functionality, 4.7 for aesthetics, 4.68 for information, and 4.1 for subjective quality. The median overall score was 4.55.

### ThessHF Usability Study

**Figure 3:**
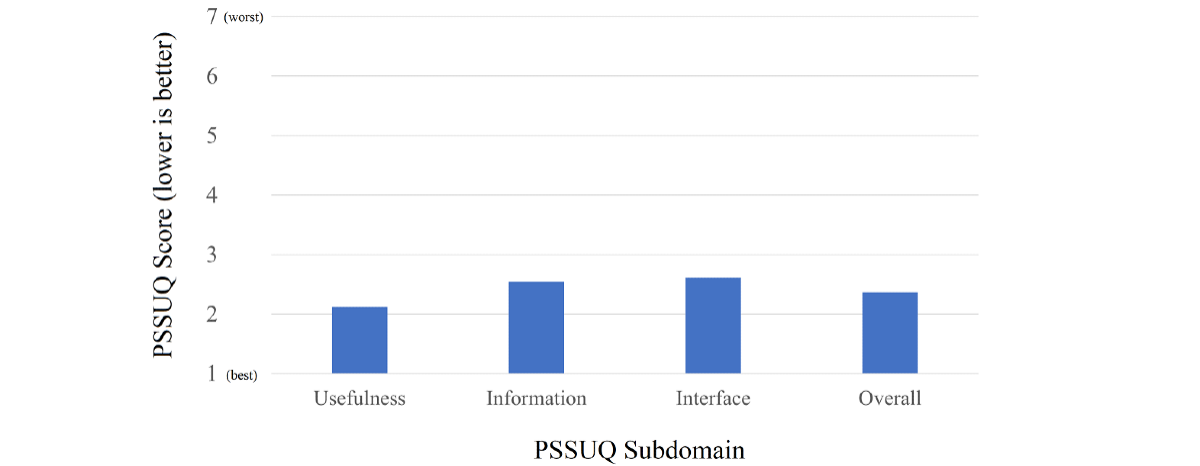
Bar chart of PSSUQ subdomain and total score. PSSUQ - Post-Study System Usability Questionnaire.

Overall, 25 patients were assessed for participating in the usability study. Among these, 11 were excluded for not owning an Android smartphone. No patient was excluded for not understanding written Greek or denying participation. In the end, 14 patients with HFrEF (mean age 64.9 [SD 9.7] years, 11 male) participated in the usability study (Table 3). The ThessHF app received an overall PSSUQ score of 2.37 (SD 0.63). In the system usefulness subdomain, the app was rated at 2.12 (SD 0.56), information quality was rated at 2.54 (SD 0.87), and the interface quality received an average score of 2.61 (SD 0.92) (Figure 3).

**Table 3:** Characteristics of patients included in the ThessHF usability.

Characteristics			Patients with heart failure with reduced ejection fraction: usability study (n=14)
Age (years), mean (SD)			64.9 (9.7)
Sex (male), n (%)			11 (79)
**New York Heart Association classification, n (%)**			
	I		1 (7)
	II		9 (64)
	III		4 (29)
Cardiac implantable electronic devices, n (%)			8 (57)
Left ventricular ejection fraction (%), mean (SD)			27.6 (8.8)
6-Minute walking distance (m), mean (SD)			465.1 (98.3)
**Questionnaire answers**			
		Smartphone ownership, n (%)	14 (100)
		Confidence in smartphone use (0-5, where 5 is the best), mean (SD)	2.89 (1.81)
		Social media use, n (%)	3 (21)
		Primary caregiver a competent smartphone user?, n (%)	12 (86)

### The Results of the THESS-HF Prospective Study

**Figure 4:**
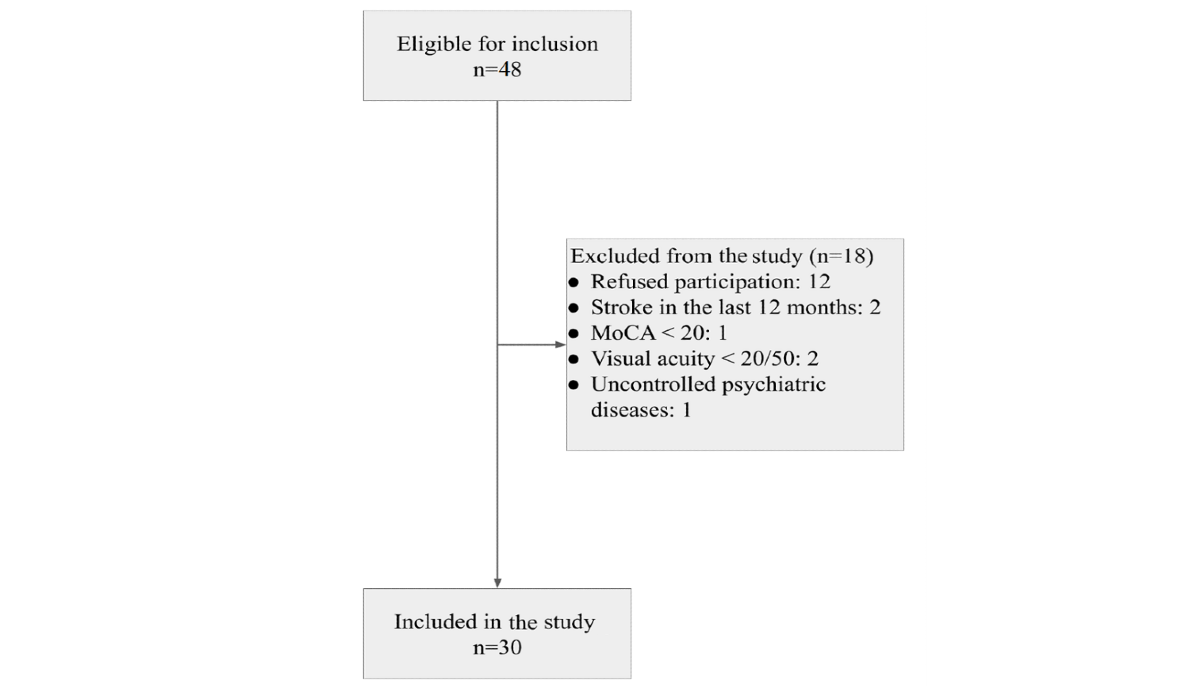
THESS-HF study patient recruitment flowchart. MoCA – Montreal Cognitive Assessment test.

A total of 30 patients were recruited in this study. The patient recruitment flowchart is presented in Figure 4. The baseline patient characteristics are tabulated in Table 4. Regarding the quality of life as measured by questionnaires KCCQ and EQ-5D-5L, a nonsignificant trend toward improvement during the study duration was observed. The mean baseline KCCQ-TTS score was 73.4 (SD 13.6), whereas the mean increase after 3 months of app usage was 5.8 (SD 15) (95% CI –0.1 to 11.6, *P*=.054). The mean baseline EQ-5D-5L VAS was 59.5% (SD 14.9%), whereas the mean increase after 3 months of app usage was 5.6% (SD 15.6%) (95% CI –0.4 to 11.5, *P*=.06). The mean quality of self-care significantly increased during the study duration, as the baseline EHFScBs score of 64.2% (SD 10.2%) increased by an average of 4.4% (SD 7.2%) (95% CI 1.7-7.1, *P*=.002). Overall, only 1 patient was hospitalized for HF decompensation during the follow-up period.

**Table 4:** Baseline characteristics of patients recruited in the THESS-HF^a^ study (n=30).

Patient parameter		Value
Age, mean (SD)		68.7 (12.4)
Male sex, n (%)		26 (87)
**Heart failure etiology, n (%)**		
	Dilatated cardiomyopathy	15 (50)
	Ischemic cardiomyopathy	15 (50)
	Hypertension	10 (33)
	Diabetes mellitus type II	12 (40)
	Coronary artery disease	17 (57)
	Atrial fibrillation	16 (53)
**Heart failure therapy at baseline, n (%)**		
	Angiotensin-converting enzyme inhibitor/angiotensin II receptor blocker	25 (83)
	Angiotensin receptor neprilysin inhibitor	15 (50)
	Beta blocker	28 (93)
	Aldosterone inhibitor	24 (80)
	Loop diuretics	21 (70)
Implanted cardiac implantable electronic device		14 (47)

^a^THESS-HF: THe Effect of a Specialized Smartphone app on Heart Failure patients’ quality of life, quality of self-care and hospitalization rate.

## Discussion

### Principal Findings

In this paper we describe the entire process of designing a smartphone-based HF intervention in an evidence-based manner. Based on our team’s thorough literature research, 4 trials [22-25] assessing apps’ effects on patient outcomes were found. Indeed, it seems that only a limited number of HF-specific apps have been tested through scientific studies [41]. All studies employed a similar type of intervention, asking patients to weigh themselves, measure their blood pressure, and assess their symptoms regularly, logging the results in an app. Patients found most features subjectively helpful, but it is unknown whether some modules are more important than others in the effort to improve patient education and self-care.

Seto et al [22] were the first to develop a smartphone app for patients with HF. Monitoring patient symptom severity, blood pressure, and body weight were basic app features. These features became a staple of patient-centered HF apps, because of their significance in the disease. A substantial increase in physician and nurse workload was a recurrent motif found in many of the examined studies [23-25]. To preserve scalability of ThessHF-based interventions, we opted against any sort of in-app communication between patients and medical personnel. As an evidence-based method of intervention, the “information–motivation–behavioral skills” model, conceptualized by Fisher et al [42] and employed by Athilingam et al [25] in their HeartMapp app, served as a robust framework that allowed us to combine the features that were included in ThessHF. Our app’s advantage over previous implementations mostly lied in its evidence-based development, as well as the innovative gamification features such as the HF knowledge quiz and participation trophies, which are expected to motivate patients maintain frequent interaction with the app.

We believe that ThessHF greatly benefited from this systematic search for app features in the literature, as well as the systematized expert input in deciding which app features should be included. Patients and mHealth experts alike rated the app positively during usability testing. Some features classified as “inclusion optional” by experts were not included. Activity tracking was decided against because of the low market penetration of wearables among the population of patients with HF. The active involvement of nursing personnel in the app was not implemented to maintain scalability in resource-scarce settings. According to the PSSUQ scores, patients found the app actually useful for their everyday self-care, while experts filling out the MARS score lauded the app’s stability and quality of information. In fact, almost all PSSUQ scores, except for the interface quality subdomain, were better than the mean ratings collected by Lewis et al [43] in a psychometric study of the questionnaire. That said, both groups found that the interface could benefit from some improvements. Thanks to input from our patients, we made several design changes to suit the app to their needs. In particular, buttons were made significantly larger and spaced out, and patients were no longer required to use the onscreen keyboard for daily use. Indeed, it is likely that designing elegant yet easy-to-use apps for patients with HF will prove a major challenge for similar mHealth interventions worldwide. As mentioned above, unstructured comments made by patients and physicians trying out ThessHF were crucial to the improvement of the graphical interface. The fact that the very physicians treating these patients with HF were tasked with actually coding the desired design changes reinforced the feeling of patients that they were an active part of the development process, increasing their motivation and bonding with the HF clinic.

The effect of app use on quality of self-care and quality of life of patients with HF was investigated in the THESS-HF pilot study. Although neither HF-specific nor health-related quality of life increased significantly during the follow-up period, the quality of self-care significantly improved. Self-care quality has been repeatedly [44-46] found to correlate with outcomes in HF. Thus, it is not unreasonable to assume that improving self-care can improve HF quality of life and morbidity in the long run. This will be tested in a multicenter prospective study aiming to recruit more patients with a longer follow-up, so as to test whether sustained use of a patient-centered HF app can yield better outcomes.

mHealth interventions are expected to become highly valuable tools in the remote care of patients with HF during the COVID-19 pandemic that started in 2019 [47]. It is thus fortuitous that innovative and noninvasive means of monitoring patients with HF have been proposed [48]. The ThessHF app proved to be a competent tool for patient support in times of this crisis. The lack of systematized patient–physician interaction or transmission of locally saved patient data through the app constituted a conscious choice to enable low physician workload and thus affordable scalability. This design decision was also advantageous insofar that patient data were never uploaded to remote databases, which would pose a significant medicolegal challenge. By contrast, physicians having direct access to patient telemetry could have further increased the app’s efficacy in improving the standard of care of patients with HF. Furthermore, usage statistics that could help highlight which features saw most use by patients as well as overall interaction time were unavailable. Our research team is actively looking into including this functionality in the future versions of the app.

As stated above, the inherent inability of data transmission through the app precluded telemetry, which could have been used to better understand different users’ engagement pattern, as well as potential technical issues that prevented patients from making full use of the app. Nonetheless, subsequent physical visits allowed us to peruse participants’ app history. This unstructured “final visit” allowed the research team to access usage statistics for some patients. Anecdotally, the research team observed that most patients replaced their handwritten arterial pressure and body weight journals with the app, a fact that kept patient interaction with the app high throughout and beyond the study duration. It should also be noted that no patient dropped out of the study, with all of them continuing some use of the app.

In many parts of the world where social distancing is sternly encouraged [35], primary care physicians and outpatient HF clinics are predicted to cease or reduce noncritical visits, whether willingly or at the behest of the local health authorities. The time originally allocated to physical visits could instead be invested in managing patients remotely, via tailor-made platforms. Indeed, now may be the time to embrace platforms with increased physician involvement, even though such attempts yielded mixed results in the past [23-25]. In this spirit, the ThessHF app is planned to incorporate remote, secure data transfer, ideally paired with wireless sensors.

In parallel with the THESS-HF study and during the lockdown imposed in Greece between March and May 2020, the Hellenic Society of Cardiology made the app available for download for all Greek patients with HF via its website, to assist them with self-care during the COVID-19 pandemic, and beyond. As of the writing of this article, 405 patients with HF downloaded the app. Because of our longstanding commitment to respect patients’ sensitive data, no analytics were available regarding app usage. Thus, the user was not asked for any data prior to downloading the app through the internet, as this would be an entirely different study.

### Conclusion

HF is a chronic disease, in which consistent and complex self-care is required to achieve good outcomes. mHealth-based interventions to improve patient education and the quality of self-care appear promising, at least in part due to the ease and low cost of their implementation [1,3,4,49]. The first step in the evidence-based development of ThessHF was a systematic review and appraisal of similar interventions. Beneficial app features were selected by a panel of physicians and implemented by physician-programmers in the beta version of the ThessHF app, which received positive reviews by patients and mHealth experts alike. In the THESS-HF study, app use correlated with improved self-care on the part of patients. A future multicenter study with longer follow-up duration will investigate whether improvements in self-care achieved through app use can lead to better outcomes for patients with HF.

## Appendix

Multimedia Appendix 1 Detailed description of the methods, PRISMA flow diagram and Cochrane's Risk of Bias table for the systematic review that was performed to find HF-oriented mHealth apps.

Multimedia Appendix 2 Screenshots of the original ThessHF app in Greek.The figure displays (a) the main screen and gamification features, (b) the actvity where patients input their blood pressure, (c) history of blood pressure available as a graph, (d) the main menu screen, where inputted parameters turn green, (e) a question out of the weekly quiz, and (f) the push notification reminding patients to take their pills.

Multimedia Appendix 3 The ThessHF app in action. The user inputs their arterial pressure, body weight and dyspnea, looks at past measurements, takes a quick quiz on HF and finally cashes in their trophies to get golden hearts.

## Abbreviations

EHFScBs: European Heart Failure Self-care Behavior Scale
HF: heart failure
HFrEF: HF with reduced ejection fraction
KCCQ: Kansas City Cardiomyopathy Questionnaire
PSSUQ: Post-Study System Usability Questionnaire
ThessHF app: The Hellenic Educational Self-care and Support Heart Failure app
THESS-HF: THe Effect of a Specialized Smartphone app on Heart Failure patients’ quality of self-care, quality of life and hospitalization rate
