# Supplementary material for: A Patient-Oriented App (ThessHF) to Improve Self-Care Quality in Heart Failure: From Evidence-Based Design to Pilot Study
Source: JMIR Mhealth Uhealth. 2021 Apr 13;9(4):e24271. doi: 10.2196/24271 (PMC8080140; doi:10.2196/24271)
Supplement: Multimedia Appendix 1 [file mhealth_v9i4e24271_app1.docx]

**Systematic Review Supplementary File**

**Methods:**

*Search strategy*

The search process was conducted in accordance with the Preferred Reporting Items for Systematic Reviews and Meta-Analyses (PRISMA) guidelines. Our team performed a thorough search of MEDLINE, EMBASE, PMC and the Cochrane Database of Systematic Reviews, in order to collect all the studies regarding mHealth administered via smartphones for HF patients in the last 10 years. All the literature research was carried out through PubMed and Google Scholar. The search terms strategy emphasized on mHealth, telemonitoring and HF.

*Inclusion criteria*

The criteria used to evaluate all the studies for eligibility were: (1) original publication reporting on the results of an experimental or semi-experimental trial, (2) publication in peer-reviewed journal, (3) publication in the last 10 years and (4) written in English. Each study was rendered eligible for inclusion after its title, abstract and full text had been examined thoroughly.

*Exclusion criteria*

The exclusion criteria were (1) studies in which the smartphone was not the primary intervention, (2) apps whose principal intended user was a health professional.

*Study selection*

Our search focused on mHealth interventions that utilized smartphone technology. Therefore, only trials in which the intervention group received a smartphone or tablet application were finally selected through the process. Trials using other forms of mobile technology, such as automated phone calls or SMSs were excluded from the final selection. Two researchers independently vetted the search results; in case of disagreement concerning the inclusion of a search result, a third independent adjudicator was called upon, whose decision informed based on the arguments of both parties and whose decision was final.

*Data extraction and quality assessment*

Each selected study was comprehensively read by the reviewer’s team in order to extract data for the characteristics of the intervention, the size of the study population, primary and secondary endpoints, especially hospitalizations and the patients’ adherence to their medical treatment.

The RCTs were assessed for their quality and potential bias with the use of the Revised Cochrane risk-of-bias tool for randomized trials (RoB 2). A complete flow chart of the review process is available below:


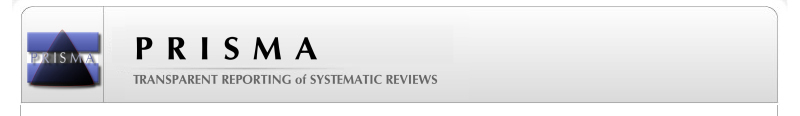
**PRISMA 2009 Flow Diagram**

Studies included in qualitative synthesis
(n = 4)

Records excluded
(n = 473)

Records screened
(n = 543)

Records after duplicates removed
(n = 1016)

## Identification

## Eligibility

## Included

## Screening

Additional records identified through other sources
(n = 18)

Records identified through database searching
(n = 2865)

Full-text articles assessed for eligibility
(n = 36)

Full-text articles excluded
Intervention didn’t use apps (n = 26)

Patient outcomes not reported (n = 4)

Intervention not specific to HF (n = 2)

**Results:**

Our search yielded 4 studies that measured the effect of app-based interventions in HF patients. Sample size was modest among all studies, varying from 18 (Athilingam et al.) to 100 (Seto et al.). The outcomes measured were similar among studies, including the number and length of readmissions for HF decompensation, the patients’ quality of life and quality of self-care, as quantified by validated questionnaires, as well as the level of adherence to medication. A complete overview of the studies is included in the main file. The results of a complete risk of bias assessment with the use of the Cochrane “Risk of bias 2.0” tool are also available below:

| **Studies:** | **Seto et al.**  **2012** | **Vuorinen et al.**  **2014** | **Hägglund et al.**  **2015** | **Athilingham et al.**  **2018** |
| --- | --- | --- | --- | --- |
| **Risk of bias arising from the randomization process** | Some concerns | High risk | Some concerns | Some concerns |
| **Risk of bias due to deviations from the intended interventions (effect of assignment to intervention)** | High risk | Some concerns | High risk | Some concerns |
| **Risk of bias due to deviations from the intended interventions (effect of adhering to intervention)** | High risk | High risk | High risk | High risk |
| **Risk of bias due to missing outcome data** | Low risk | Low risk | Low risk | Low risk |
| **Risk of bias in measurement of the outcome** | Low risk | Low risk | Low risk | Low risk |
| **Risk of bias in selection of the reported result** | Low risk | Low risk | Some concerns | Some concerns |

**Table 2:** Risk of bias assessment based on the modified risk of bias Cochrane collaboration tool (RoB 2).
