# Supplementary figures and images for: A Patient-Oriented App (ThessHF) to Improve Self-Care Quality in Heart Failure: From Evidence-Based Design to Pilot Study
Source: JMIR Mhealth Uhealth. 2021 Apr 13;9(4):e24271. doi: 10.2196/24271 (PMC8080140; doi:10.2196/24271)

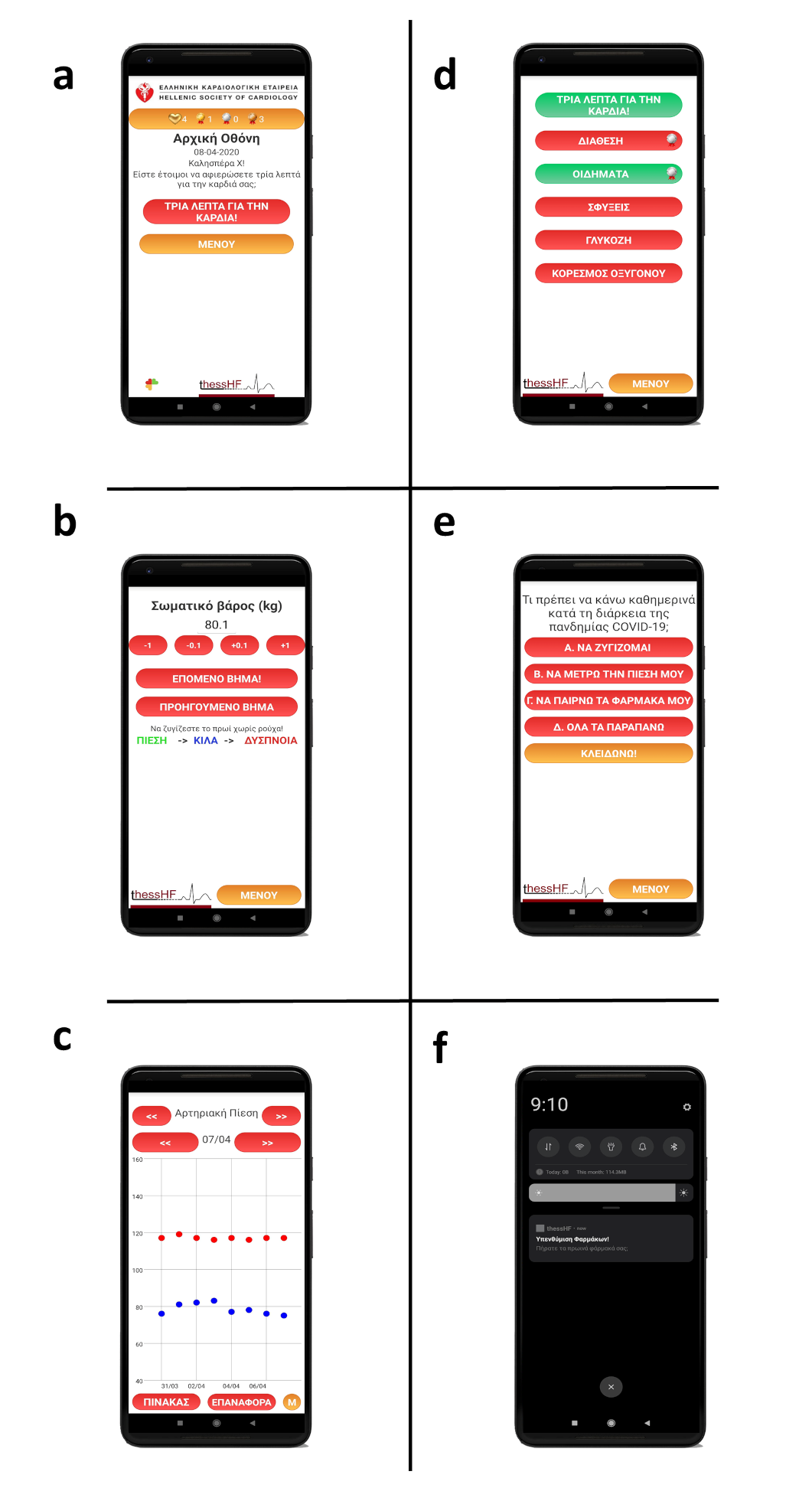

Supplement: Multimedia Appendix 2 [file mhealth_v9i4e24271_app2.docx]
